# Mitochondrial DNA structure in the Arabian Peninsula

**DOI:** 10.1186/1471-2148-8-45

**Published:** 2008-02-12

**Authors:** Khaled K Abu-Amero, José M Larruga, Vicente M Cabrera, Ana M González

**Affiliations:** 1 Mitochondrial Research Laboratory, Department of Genetics, King Faisal Specialist Hospital and Research Center, Riyadh, Saudi Arabia; 2 Department of Genetics, Faculty of Biology, University of La Laguna, Tenerife 38271, Spain

## Abstract

**Background:**

Two potential migratory routes followed by modern humans to colonize Eurasia from Africa have been proposed. These are the two natural passageways that connect both continents: the northern route through the Sinai Peninsula and the southern route across the Bab al Mandab strait. Recent archaeological and genetic evidence have favored a unique southern coastal route. Under this scenario, the study of the population genetic structure of the Arabian Peninsula, the first step out of Africa, to search for primary genetic links between Africa and Eurasia, is crucial. The haploid and maternally inherited mitochondrial DNA (mtDNA) molecule has been the most used genetic marker to identify and to relate lineages with clear geographic origins, as the African Ls and the Eurasian M and N that have a common root with the Africans L3.

**Results:**

To assess the role of the Arabian Peninsula in the southern route, we genetically analyzed 553 Saudi Arabs using partial (546) and complete mtDNA (7) sequencing, and compared the lineages obtained with those present in Africa, the Near East, central, east and southeast Asia and Australasia. The results showed that the Arabian Peninsula has received substantial gene flow from Africa (20%), detected by the presence of L, M1 and U6 lineages; that an 18% of the Arabian Peninsula lineages have a clear eastern provenance, mainly represented by U lineages; but also by Indian M lineages and rare M links with Central Asia, Indonesia and even Australia. However, the bulk (62%) of the Arabian lineages has a Northern source.

**Conclusion:**

Although there is evidence of Neolithic and more recent expansions in the Arabian Peninsula, mainly detected by (preHV)1 and J1b lineages, the lack of primitive autochthonous M and N sequences, suggests that this area has been more a receptor of human migrations, including historic ones, from Africa, India, Indonesia and even Australia, than a demographic expansion center along the proposed southern coastal route.

## Background

The hypothesis that modern humans originated in Africa and later migrated out to Eurasia replacing there archaic humans [1,2] has continued to gain support from genetic contributions [3-6]. Anthropologically, the most ancient presence of modern humans out of Africa has been documented in the Levant about 95–125 kya [7,8], and in Australia about 50–70 kya [9]. Based on archaeological [10] and classic genetic studies [11,12] two dispersals from Africa were proposed: A northern route that reached western and central Asia through the Near East, and a Southern route that, coasting Asia, reached Australia. However, ages for these dispersals were very tentative. The first phylogeographic analysis using complete mtDNA genomic sequences dated the out of Africa migrations around of 55–70 kya, when two branches, named M and N, of the African macrohaplogroup L3 radiation supposedly began the Eurasian colonization [5,6]. A more recent analysis, based on a greater number of sequences, pushed back the lower bound of the out-of-Africa migration, signed by the L3 radiation, to around 85 kya [13]. This date is no so far from the above commented presence of modern humans in the Levant about 100–125 kya. Interestingly, this migration is also in frame with the putative presence of modern humans in Eritrean coasts [14], and corresponds with an interglacial period (OIS 5), when African faunas expanded to the Levant [15]. After that, it seems that, at least in the Levant, there was a long period of population bottleneck, as there is no modern human evidence in the area until 50 kyr later, again in a relatively warm period (OIS 3). This contraction phase might be reflected in the basal roots of M and N lineages by the accumulation of 4 and 5 mutations before their next radiation around 60 kya [13].

**Figure 1:**
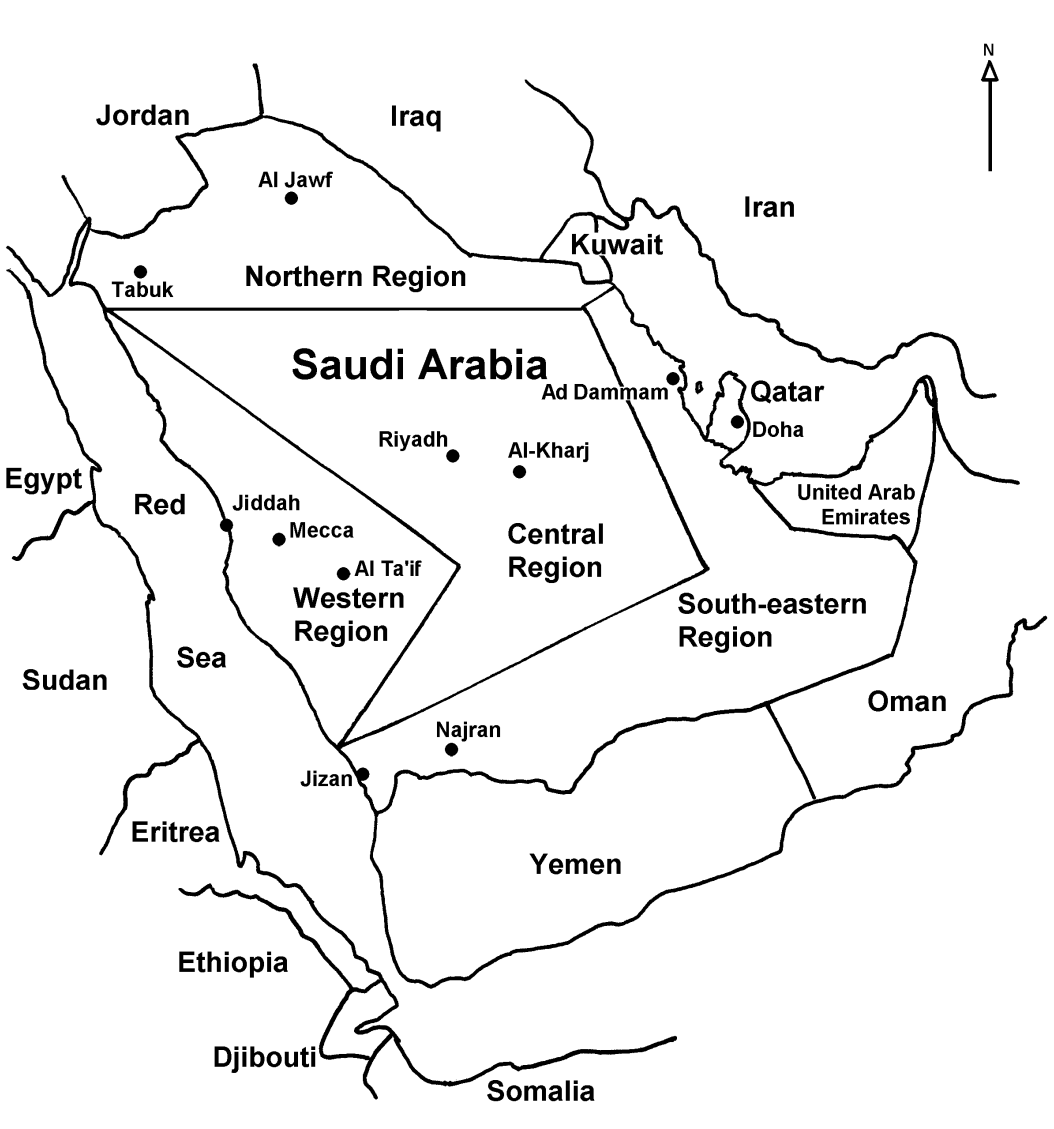
Map of the Arabian Peninsula showing the Saudi regions and Arabian countries studied.

Paradoxically this expansion began in a glacial period (OIS 4). At glacial stages it is supposed that aridity in the Levant was a strong barrier to human expansions and that an alternative southern coastal route, crossing the Bab al Mandab strait to Arabia, could be preferred. Consequently, based on the phylogeographic distribution of M and N mtDNA clusters, with the latter prevalent in western Eurasia and the former more frequent in southern and eastern Asia, it was proposed that two successive migrations out of Africa occurred, being M and N the mitochondrial signals of the southern and northern routes respectively [6]. Furthermore, the star radiation found for the Indian and East Asian M lineages was taken as indicative of a very fast southern dispersal [6]. However, posterior studies revealed the presence of autochthonous M and N lineages all along the southern route, from South Asia [16-21], through Malaysia [13] and to Near Oceania and Australia [22-26]. Accordingly, it was hypothesized that both lineages were carried out in a unique migration [27,28], and even more, that the southern coastal trail was the only route, being the western Eurasian colonization the result of an early offshoot of the southern radiation in India [29,13]. Under these suppositions, the Arabian Peninsula, as an obliged step between East Africa and South Asia, has gained crucial importance, and indeed several mtDNA studies have recently been published for this region [30-32]. However, it seems that the bulk of the Arab mtDNA lineages have northern Neolithic or more recent Asian or African origins. Although a newly defined clade L6 in Yemenis, with no close matches in the extant African populations, could suggest an ancient migration from Africa to Yemen [30], the lack of N and/or M autochthonous lineages left the southern route without genetic support. It could be that unfavorable climatic conditions forced a fast migration through Arabia without leaving a permanent track, but it is also possible that sample sizes have been insufficient to detect ancient residual lineages in the present day Arab populations. To deal with this last possibility we have enlarged our previous sample of 120 Saudi Arabs [31] to 553, covering the main regions of this country (Figure 1). In this sample we sequenced the non-coding HVSI and HVSII mtDNA regions and unequivocally assorted the obtained haplotypes into haplogroups analyzing diagnostic coding region positions by restriction fragment length polymorphisms (RFLP) or fragment sequencing. Furthermore, when rare haplotypes were found, we carried out genomic mtDNA sequencing on them. In addition, the regional subdivision of the Saudi samples and the analysis of the recently published mtDNA data for Yemen [30] and for Yemen, Qatar, UAE and Oman [32] allowed us to asses the population structure of the Arabian Peninsula and its relationships with surrounding populations.

## Results

A total of 365 different mtDNA haplotypes were observed in 553 Saudi Arab sequences. 299 of them (82%) could have been detected using only the HVSI sequence information and 66 (18%) when the HVSII information was also taken into account. Additional analysis of diagnostic positions allowed the unequivocal assortment of the majority (96%) of the haplotypes into subhaplogroups [see Additional file 1]. However, 11 haplotypes were classified at the HV/R level, 3 assigned to macrohaplogroups L3*, M* and N* respectively, and only one was left unclassified [see Additional file 1]. The most probable origin of these Saudi haplotypes deserves a more detailed analysis.

### Macrohaplogroup L lineages

Sub-Saharan Africa L lineages in Saudi Arabia account for 10% of the total. χ^2 ^analyses showed that there is not significant regional differentiation in this Country. However, there is significant heterogeneity (p < 0.001) when all the Arabian Peninsula countries are compared. This is mainly due to the comparatively high frequency of sub-Saharan lineages in Yemen (38%) compared to Oman-Qatar (16%) and to Saudi Arabia-UAE (10%). Most probably, the higher frequencies shown in southern countries reflect their greater proximity to Africa, separated only by the Bab al Mandab strait. However, when attending to the relative contribution of the different L haplogroups, Qatar, Saudi Arabia and Yemen are highly similar for their L3 (34%), L2 (36%) and L0 (21%) frequencies whereas in Oman and UAE the bulk of L lineages belongs to L3 (72%). In this enlarged sample of Saudi Arabs, representatives of all the recently defined East African haplogroups L4 [30], L5 [33], L6 [30] and L7 [34], have been found. The only L4 Saudi haplotype belongs to the L4a1 subclade defined by 16207T/C transversion. Although it has no exact matches its most related types are found in Ethiopia [30]. Four L5 lineages have been found in Saudi Arabia but all have the same haplotype that belongs to the L5a1 subclade defined in the HVSI region by the 16355–16362 motif [30]. It has matches in Egypt and Ethiopia. L6 was found the most abundant clade in Yemen [30]. It has been now detected in Saudi Arabia but only once. This haplotype (16048-16223-16224-16243-16278-16311) differs from all the previous L6 lineages by the presence of mutation 16243. In addition it lacks the 16362 transition that is carried by all L6 lineages from Yemen but has the ancestral 16048 mutation only absent in one Yemeni lineage [30]. This Saudi type adds L6 variability to Arabia, because until now L6 was only represented by a very abundant and a rare haplotype in Yemen. Attending to the most probable geographic origin of the sub-Saharan Africa lineages in Saudi Arabia, 33 (61%) have matches with East Africa, 7 (13%) with Central or West Africa whereas the rest 14 (26%) have not yet been found in Africa. Nevertheless, half of them belong to haplogroups with Western Africa origin and the other half to haplogroups with eastern Africa adscription [35,30]. It is supposed that the bulk of these African lineages reached the area as consequence of slave trade, but more ancient historic contacts with northeast Africa are also well documented [36,30,31].

### Macrohaplogroup M lineages

M lineages in Saudi Arabia account for 7% of the total. Half of them belong to the M1 African clade. There is no significant heterogeneity within Saudi Arabia regions nor among Arabian Peninsula countries for the total M frequency. However, when we compared the frequency of the African clade M1 against that of the other M clades of Asiatic provenance, it was significantly greater in western Arabian Peninsula regions than in the East (χ^2 ^= 12.53 d.f. = 4 p < 0'05).

#### Inclusion of rare Saudi and other published African M1 sequences into the M1 genomic phylogenetic tree

**Figure 2:**
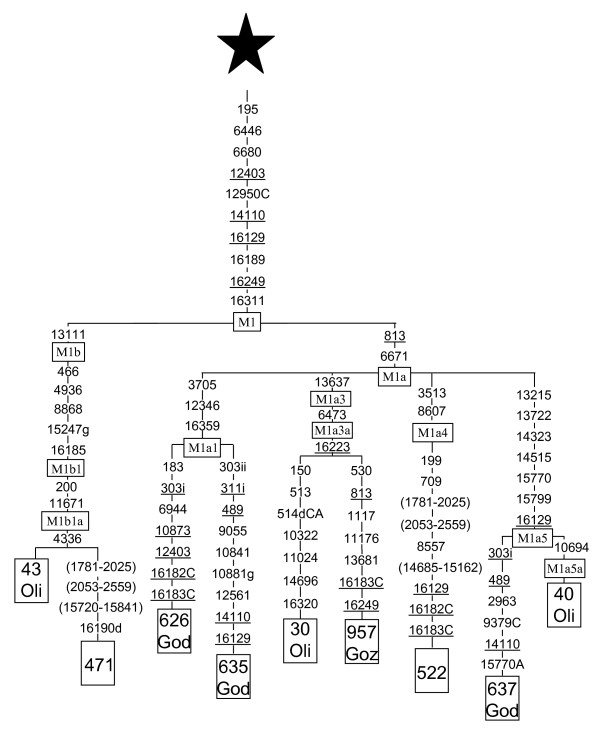
**Phylogenetic tree based on complete M1 sequences**. Numbers along links refer to nucleotide positions. A, C, G indicate transversions; "d" deletions and "i" insertions. Recurrent mutations are underlined. Regions not analyzed are in parenthesis. Star differs from rCRS [62, 63] at positions: 73, 263, 303i, 311i, 489, 750, 1438, 2706, 4769, 7028, 8701, 8860, 9540, 10398, 10873, 10400, 11719, 12705, 14766, 14783, 15043, 15301, 15326, 16223 and 16519. GenBank accession numbers of the subjects retrieved from the literature are: 43 Oli (EF060354), 30 Oli (EF060341) and 40 Oli (EF060351) from Olivieri et al. [37]; 626 God (EF184626), 635 God EF184635 and 637 God (EF184637) from Gonder et al. [39]; and 957 Goz (DQ779926) from González et al. [38].

Recent phylogenetic and phylogeographic analysis of this haplogroup [30,37,38] have suggested that the M1a1 subclade (following the nomenclature of Olivieri et al. [37]), is particularly abundant and diverse in Ethiopia and M1b in northwest Africa and the European and African Mediterranean areas. Other M1a subclades have a more generalized African distribution. Half of the M1 lineages in Saudi Arabia belong to the Ethiopian M1a1 subclade and the same proportion holds for other Arabian Peninsula countries [30,32]. However, as a few M1 haplotypes did not fit in the M1a1 cluster we did genome sequencing for two of them (Figure 2). Lineage 471 resulted to be a member of the North African clade M1b, more specifically to the M1b1a branch. As we have detected another M1b lineage in Jordan [38], it is possible that the Saudi one could have reached Arabia from the Levant or from northwest African areas. The second Saudi lineage (522) belongs to a subcluster (M1a4) that is also frequent in East Africa [37]. Recently, Tanzanian lineages have been studied by means of complete mtDNA sequences [39]. Three of these sequences also fall into the M1 haplogroup. Two of them belong to the Ethiopian M1a1 subclade (God 626 and God 635), and the third (God637) shares the entire motif that characterizes lineage M1a5 [37] with the exception of transition 10694. Therefore, this mutation should define a new subcluster M1a5a (Figure 2). The lineages found in Tanzania further expand, southeastwards, the geographic range of M1 in sub-Saharan Africa. Inspecting the M1 phylogeny of Olivieri et al. [37] we realized that our lineage 957 [38] has the diagnostic positions 13637, that defines M1a3 and 6463 that defines the M1a3a branch. Therefore, we have placed it as an M1a3a lineage with an 813 retromutation (Figure 2). It seems that, likewise L lineages, the M1 presence in the Arabian Peninsula signals a predominant East African influence with possible minor introductions from the Levant.

#### Inclusion of rare Saudi Asiatic M sequences into the macrohaplogroup M tree

**Figure 3:**
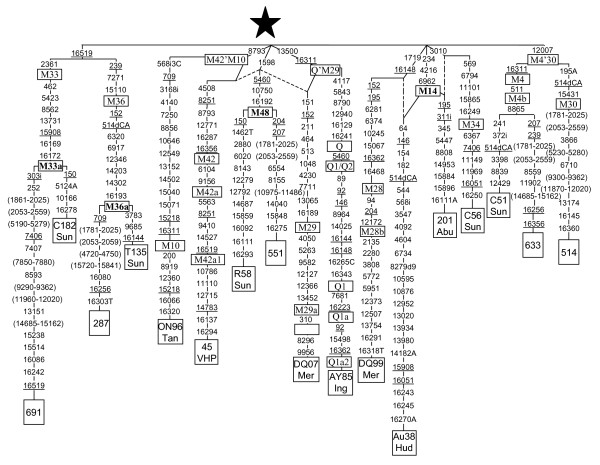
**Phylogenetic tree based on complete M sequences**. Numbers along links refer to nucleotide positions. A, C, T indicate transversions; "d" deletions and "i" insertions. Recurrent mutations are underlined. Regions not analyzed are in parenthesis. Star differs from rCRS [62, 63] at positions: 73, 263, 303i, 311i, 489, 750, 1438, 2706, 4769, 7028, 8701, 8860, 9540, 10398, 10873, 10400, 11719, 12705, 14766, 14783, 15043, 15301, 15326, 16223 and 16519. GenBank accession numbers of the subjects retrieved from the literature are: C182 Sun (AY922276), T135 Sun (AY922287), R58 Sun (AY922299), C56 Sun (AY922274), and C51 Sun (AY922261) from Sun et al. [20]; ON96 Tan (AP008599) from Tanaka et al. [40]; 45 VHP (DQ404445) from van Holst Pellekaan et al. [25]; DQ07 Mer (DQ137407), DQ99 Mer (DQ137399) from Merriwether et al. [24]; AY85 Ing (AY289085) from Ingman and Gyllensten [22]; Au38 Hud (EF495222) from Hudjashov et al. [26]; and 201 Abu (DQ904234) from Abu-Amero et al. [31].

The majority (12) of the 19 M lineages found in the Arabian Peninsula that do not belong to M1 [see Additional file 1] have matches or are related to Indian clades, which confirm previous results [30,31]. In addition, in this expanded Saudi sample, we have found some sequences with geographic origins far away from the studied area. For instance, lineage 569 [see Additional file 1] has been classified in the Eastern Asia subclade G2a1a [40] but probably it has reached Saudi Arabia from Central Asia where this branch is rather common and diverse [41]. Indubitably the four sequences (196, 479, 480 and 494) are Q1 members and had to have their origin in Indonesia. In fact their most related haplotypes were found in West New Guinea [42]. All these sequences could have arrived to Arabia as result of recent gene flow. Particularly documented is the preferential female Indonesian migration to Saudi Arabia as domestic workers [43]. Five undefined M lineages were genome sequenced (Figure 3). It is confirmed that 5 of the 6 Saudi lineages analyzed have also Indian roots. Lineage 691 falls into the Indian M33 clade because it has the diagnostic 2361 transition. In addition, it shares 7 transitions (462, 5423, 8562, 13731, 15908, 16169, 16172) with the Indian lineage C182 [20], which allows the definition of a new subclade M33a. Lineage 287 is a member of the Indian M36 clade because it possesses its three diagnostic mutations (239, 7271, 15110). As it also shares 8 additional positions with the Indian clade T135 [20], both conform an M36a branch (Figure 3). Saudi 514 belongs to the Indian clade M30 as it has its diagnostic motif (195A-514dCA-12007-15431). Lineage 633 also belongs to the related Indian clade M4b defined by transitions 511, 12007 and 16311. In addition it shares mutation 8865 with the C51 Indian lineage [20] that could define a new M4b2 subclade. We have classified sequence 551 as belonging to a new Indian clade M48 defined by a four transitions motif (1598-5460-10750-16192) which is shared with the M Indian lineage R58 (Figure 3). Australian clade M42 [44] and New Britain M29 clade [24] also have 1598 transition as a basal mutation. However, they are respectively more related to the East Asia clade M10 [40] and to the Melanesian Q clade [27], as their additionally shared basal mutations are less recurrent than transition1598 [45]. All these Indian M sequences have been found in Arabia as isolated lineages that belong to clusters with deep roots and high diversity in India. Therefore, its presence in Arabia is better explained by recent backflow from India than by supposing that these lineages are footsteps of an M ancestral migration across Arabia.

The Saudi sequence 201 deserves special mention (Figure 3). It was previously tentatively related to the Indian M34 clade because both share the 3010 transition. However, it was stated that due to the high recurrence of 3010 most probably the 201 sequence would belong to a yet undefined clade [31]. The recent study of new Australian lineages [26] has allowed us to find out an interesting link between their Australian M14 lineage and our Saudi 201 sequence (Figure 3). The authors related M14 to the Melanesian clade M28 [24] because both share the 1719–16148 motif [26]. We think that the alternative motif shared with the Saudi lineage, 234-4216-6962, (Figure 3) is stronger, as 1719 and 16148 transitions are more recurrent than 234, 4216 and 6962 [45]. Therefore, we think that the last three mutations defined the true root of the Australian M14 clade and relate it to a Saudi Arab sequence.

### Macrohaplogroup N lineages

All the main non R West Eurasian haplogroups that directly spread from the root of macrohaplogroup N (N1a, N1b, N1c, I, W, X) were present in Saudi Arabia albeit in low frequencies [see Additional file 2]. Summing up, their total frequency only represents 12% of the Saudi pool. There is no heterogeneity among Saudi regions for the joint distribution of these haplogrups, which is in contrast with the high differentiation observed (χ^2 ^= 13.9; d.f. 3; p < 0.01) when all the Arabian Peninsula countries were compared. However, this is mainly due to the high frequency in UAE of unclassified N* lineages [32]. The three N1 subgroups have similar frequencies (2.4%) in Saudi Arabia [see Additional file 2], but haplotypic diversity (h) reach different levels being comparatively high for N1b (0.89) or N1a (0.83) and lower for N1c (0.63). N1a is also very diverse (0.89) in Yemen [30] but there are no haplotypic matches between both Arabian countries, and only one (16147G-16172-16223-16248-16355) of the 7 Saudi haplotypes has an exact match in Ethiopia. The high diversity of N1a in the Arabian Peninsula, Ethiopia and Egypt raises the possibility that this area was a secondary center of expansion for this haplogroup. However, the highest diversity for N1b and N1c are in Turkey, and Kurds and Iranians, respectively [see Additional file 3]. Only haplogroup N1c shows significant (χ^2 ^= 12.8; d.f. 3; p < 0.01) regional distribution in Saudi being the highest frequencies in the Northern region [see Additional file 2]. Haplogroup I has been only detected in Central and Southeastern regions in Saudi Arabia and in all Arabian Peninsula countries with the exception of UAE [32]. Haplogroup W has not been found in the western Saudi region nor in the Southern Arabian Yemen and Oman countries [30,32]. Finally, haplogroup X is present in all the Saudi regions and in all Arabian countries excepting Oman [30,32]. Two geographically well differentiated X branches can be distinguished by HVSII positions [46]. The North African specific subclade X1 was defined by the 146 transition and the Eurasian specific subclade X2 by the 195 transition. As it was already found in Yemen [30], all the X haplotypes found in Saudi Arabia have the 195 transition, falling within the Eurasian branch, which discards East African introductions. Curiously, only the basic haplotype (16189-16223-16278) has matches with other regions.

### Macrohaplogroup R lineages

Macrohaplogroup R is the main branch of N and their major subclades (H, J-T, K-U) embraced the majority of the West Eurasian mtDNA lineages. In Saudi Arabia R derivates represent a (70.5 ± 2.4) % of the total having a homogeneous regional distribution. This contrasts with the significant heterogeneity (χ^2 ^= 46.1; d.f. 4; p < 0.001) found for the whole Arabian Peninsula, although it is due to the low frequency of R in Yemen (46.2%). The Western Asia haplogroup H is the most abundant haplogroup in Europe and the Near East [47]. However in the Arabian Peninsula its mean frequency (9.4 ± 1.1) is moderate, reaching its highest value in Oman (13.3%) and its lowest, again, in Yemen (7.2%). Within Saudi Arabia, the highest frequencies are in the Northern and Central regions (9.3%) and the lowest in the West region (2.8%), being CRS, H2a1 and H6 the most abundant subclades which confirm other authors results [48]. Haplogroup T shows regional heterogeneity in Saudi Arabia (χ^2 ^= 10.4; d.f. 3; p < 0.05) and has significantly lower frequencies in Southern Yemen and Oman countries (χ^2 ^= 5.8; p < 0.05). Furthermore, whereas subclade T3 is the most abundant in the Saudi Central region, subclades T1 and T5 are so in Northern and Western regions. Haplogroup U comprises numerous branches (U1 to U9 and K) that have different geographic distributions [47,16,17,49]. In Saudi Arabia all of them have representatives albeit in minor frequencies, K (4%) and U3 (2.3%) being the most abundant clades. There is no geographical heterogeneity for the total U distribution in Saudi Arabia. Nevertheless, it is significantly different among the Arabian Peninsula countries (χ^2 ^= 12.0; d.f. 4; p < 0.05), with Southern countries showing higher frequencies than the others [see Additional file 3]. Analysis of specific haplogroups reveals some interesting geographic distributions in the Arabian Peninsula. The European clade U2e and the rare clade U9 (χ^2 ^= 10.5; d.f. 4; p < 0.05) could have reached the Arabian Peninsula from northern areas. On the contrary, the Indian clade U2 (χ^2 ^= 34.5; d.f. 4; p < 0.001), U3, U4, U7 (χ^2 ^= 11.7; d.f. 4; p < 0.05) and K (χ^2 ^= 10.5; d.f. 4; p < 0.05), most probably came from the East. Finally, the North African clade U6, had a Western provenance. Haplogroup (preHV)1 was phylogenetically and phylogeographically studied in detail previously [31]. However, this enlarged Saudi sample has allowed a regional analysis in Saudi Arabia. An heterogeneity test showed that (preHV)1 is significantly (χ^2 ^= 8.5; d.f. 3; p < 0.05) more abundant in Northern (18.6%) and Central (21.8%) regions, and has particularly low frequency in the West region (8.3%). Extending the analysis to the whole Arabian Peninsula the heterogeneity grows considerably (χ^2 ^= 30.9; d.f. 4; p < 0.001). This is mainly due to the comparatively lower frequencies in Yemen, Qatar and UAE [see Additional file 3]. Most probably, this haplogroup reached Arabia from the North [31], extending its geographic range to the South using mainly internal instead of coastal routes. As a whole, haplogroup J reaches its highest frequency in Saudi Arabia [31], where its regional distribution is also significantly heterogeneous (χ^2 ^= 15.0; d.f. 3; p < 0.01), but opposite to that found for (preHV)1. For the J, the West (37.5%) and Southeast (25.7%) regions have higher frequencies than the Central (17.6%) and North (16.3%) regions. Heterogeneity in the whole Peninsula is also significant (χ^2 ^= 16.5; d.f. 4; p < 0.01) being Saudi Arabia (21%) and Qatar (17.8%) the two countries with the highest J frequencies. However, the subclade distribution is different in each country. Subclade J1b is the main contributor (9.4%) in Saudi Arabia while other J subclades account for 14.5% in Qatar [see Additional file 3]. With the Qatar exception, J1b is the most frequent subclade in the Arabian Peninsula [see Additional file 3]. So for, it deserves more detailed phylogenetic and phylogeographic studies.

### Phylogeny of haplogroup J1b

**Figure 4:**
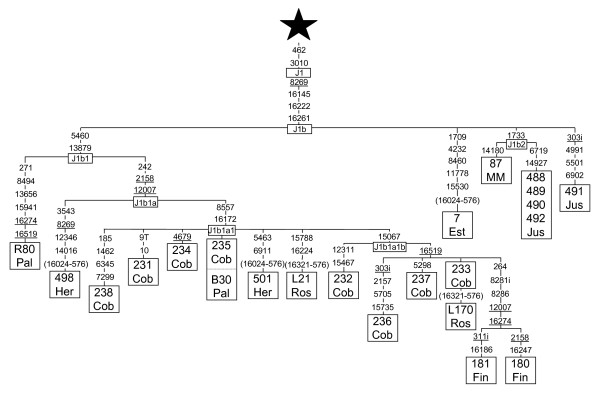
**Phylogenetic tree based on complete J1b sequences**. Numbers along links refer to nucleotide positions. T indicates transversion and "i" insertions. Recurrent mutations are underlined. Regions not analyzed are in parenthesis. Star differs from rCRS [62, 63] at positions: 73, 263, 295, 311i, 489, 750, 1438, 2706, 4216, 4769, 7028, 8860, 10398, 11251, 11719, 12612, 13708, 14766, 15326, 15452A, 16069 and 16126. GenBank accession numbers of the subjects retrieved from the literature are: R80 Pal (AY714033) and B30 Pal (AY714035) from Palanichamy et al. [18]; 498 Her (EF657673) and 501 Her (EF657678) from Herrnstad et al. [64]; 238 Cob (AY495238), 231 Cob (AY495231), 234 Cob (AY495234), 235 Cob (AY495235), 232 Cob (AY495232), 236 Cob (AY495236), 237 Cob (AY495237) and 233 Cob (AY495233) from Coble et al. [65]; L21 and L170 from Rose et al. [66]; 181 Fin (AY339582) and 180 Fin (AY339581) from Finnilä et al. [50]; 7 Est from Esteitie et al. [67]; 87 MM (AF381987) from Maca-Meyer et al. [6]; 488 (DQ282488), 489 (DQ282489), 490 (DQ282490), 492 (DQ282492) and 491 Jus (DQ282491) from Just et al. [68].

We have used 23 haplogroup J complete sequences to construct a refined J1b phylogeny. As a subclade of J1, J1b is characterized by transition 462 and 3010 [50]. In addition, J1b is defined by transition 8269 in the coding region and by the HVSI motif, 16145-16222-16261 (Figure 4). Transitions 5460 and 13879 are now diagnostic of the J1b1 subclade, previously named J1b [47,18]. In addition, the 242-2158-12007 motif defines a J1b1a branch. Furthermore, sequences carrying transitions 8557 and 16172 cluster now into the J1b1a1 clade formerly named J1b1 [47,18]. Finally, transition 15067 defines an additional J1b1a1b group (Figure 4). In order to accurately incorporate our previously published J1b Moroccan sequence [6] into the present phylogeny, we have re-sequenced all the fragments comprising any mutation present in related branches. Only transition 8269 was overlooked in our anterior analysis. This Moroccan sequence shares the 1733 transition with four Hispanic sequences defining a new J1b2 clade (Figure 4). Using time for the most recent common ancestor (TMRCA) as an upper bound for a cluster radiation, we estimated a Paleolithic time of 29,040 ± 8,061 years for the entire J1b clade and a Neolithic age of 9,175 ± 3,092 years for the J1b1a1 subclade.

### Phylogeography of haplogroup J1b

**Figure 5:**
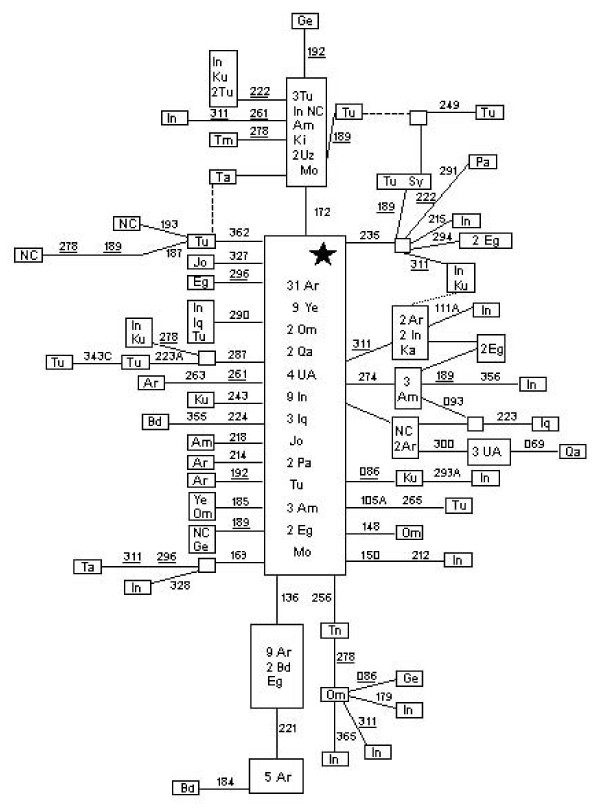
**Reduced median network relating J1b HVSI sequences**. The central motif (star) differs from rCRS at positions: 16069 16126 16145 16222 16261 for HVI control region. Numbers along links refer to nucleotide positions minus 16000: homoplasic mutations are underlined. The broken lines are less probable links due to mutation recurrence. Size of boxes is proportional to the number of individuals included. Codes are: Am = Armenian, Ar = Saudi Arab, Bd = Bedouin Arab, Eg = Egyptian, Ge = Georgian, In = Iranian, Iq = Iraqi, Jo = Jordanian, Ka = Kazakhs, Ki = Kirghiz, Ku = Kurd, Mo = Moroccan Berber, NC = Caucasian, Om = Omani, Pa = Palestinian, Qa = Qatar, Sy = Syrian, Ta = Tajik, Tm = Turkmen, Tn = Tunisian, Tu = Turk, UA = United Arab Emirates, UZ = Uzbek, Ye = Yemeni.

Figure 5 shows the reduced median network obtained from 173 J1b HVSI based haplotypes, found in a search of 6665 HVSI sequences belonging to the Arabian Peninsula and the surrounding Near East, Caucasus-Caspian, and North and Northeast African regions [see Additional files 4 and 5]. The basic central motif (16069-16126-16145-16222-16261) is the most abundant and widespread haplotype in the four areas. In Saudi Arabia this motif is particularly abundant being responsible of the relative low variability of J1b in this country (Table 1). The radiation of this clade widely affected the five studied areas and extended to the European and North African countries of the Mediterranean basin although in low frequencies. One of the main offshoots of the J1b radiation is the J1b1a1 subclade characterized in the HVSI region by the 16172 transition. It is widespread in the Near East, the Caucasus, and Northern and Central Europe where its diversity is the highest (Table 1). However it has not been detected in the Arabian Peninsula. It seems therefore that the first J1b radiation mainly affected southern countries whereas secondary spreads reached northern areas probably due to better climatic conditions. At this respect it is worth mentioning that a subsequent J1b1a1 radiation characterized by the basic 16192 transition (J1b1a1a subclade) had a northwest European expansion being a Georgian and a Russian its only detected outsiders [51]. In addition, several J1b expansions, as those rooted by the 16235 and the 16287 transitions, occurred in the Near East; whereas others, as those represented by the 16093 and the 16136 basic transitions, were mainly confined to the Arabian Peninsula (Figure 5). The TMRCA for the whole J1b haplogroup based on HVSI sequences was of 19,480 ± 4,119 years, which is more in accordance with the age of the group obtained from complete sequences and applying the Ingman et al. [5] mutation rate (21,524 ± 5,974) than with the previously cited estimation based on that of Mishmar et al. [52]. However, in all cases this radiation is placed in the Paleolithic. On the contrary, the HVSI based age for J1b1a1 (10,621 ± 4,982) is closer to that obtained following Mishmar et al. [52] (9,175 ± 3,092) than that obtained (6,800 ± 2,292) according to Ingman et al. [5]. For the age calculation of the Arab 16136 branch, two Yemeni Jews that shared the 16069-16126-16136-16145-16221 haplotype [47] were included. The age obtained (11,099 ± 8,381 years) is similar to that calculated for the northern J1b1a1 subclade, pointing to a simultaneous spread of different J1b branches in different geographic areas most probably due to generalized mild climatic conditions.

**Table 1:** K haplotype diversity (in %) and π (× 1000) diversity for the total J1b, J1b1a1 and J1b1a1a subhaplogroups.

		haplotypes	sample	K	π
J1b	Ara-tot	15	81	19	3.59 ± 2.65
	Cau-tot	14	23	**61**	7.91 ± 4.98
	NE-tot	29	56	52	**8.67 **± 5.23
	Eu-tot	35	112	31	6.32 ± 4.02

J1b1a1	Ara-tot	-	**0**	-	
	Cau-tot	4	8	**50**	2.50 ± 2.33
	NE-tot	4	10	40	3.78 ± 3.02
	Eu-tot	20	88	23	**3.97 **± 2.84

J1b1a1a	Ara-tot	-	**0**	-	-
	Cau-tot	1	**1**	100	-
	NE-tot	-	**0**	-	-
	**Eu-tot**	7	41	17	1.59 ± 1.56

### Population based comparisons

**Figure 6:**
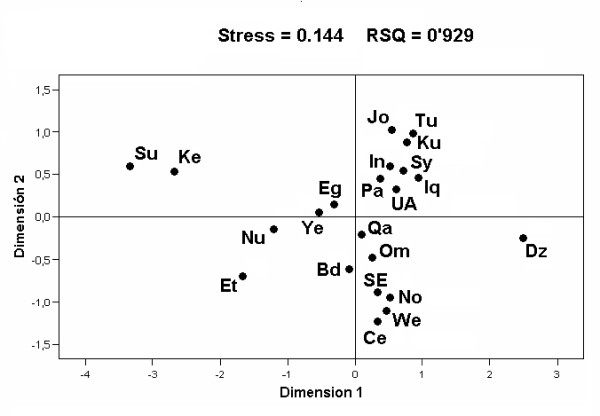
**Graphical relationships among the studied populations**. MDS plot based on F_ST _haplogroup distances. Codes are: Ce = Central Saudi Arab, Dz = Druze, Et = Ethiopian, Ke = Kenyan, No = Northern Saudi Arab, Nu = Nubian, SE = Southeastern Saudi Arab, Su = Sudan, We = Western Saudi Arab. Others codes as in Figure 5.

In order to assess the degree of regional differentiation in Saudi Arabia we performed AMOVA analyses, based on haplogroup (p < 0.001) and haplotypic (p < 0.05) frequencies. They showed significant inter regional variability, mainly due to the heterogeneous composition of the Central Region. Haplogroup frequency differentiation is also found when all the Arabian Peninsula countries were taken into account (p < 0.01). In spite of this, when the Arabian Peninsula samples were compared with those of surrounding African, Near East and Caucasus areas by means of pair-wise F_ST _distances, based on haplogroup frequencies, and their relationships graphically represented (Figure 6), it is worth mentioning that all the Saudi samples, including a small Bedouin sample [53], closely cluster together. However, two Arabian Peninsula countries, Yemen and UAE, showed marginal positions. The first, due to its greater frequency of African L haplogroups, congruently approaches Egypt and Nubian, whereas the second, due to the relative scarcity of this African component and the greater contribution of Eurasian clades (as HV, some T subgroups, and the whole U haplogroup, excepting U6) to its mtDNA pool, is placed in close proximity to the Near East samples (Figure 6).

## Discussion

### Eurasian and African influences in the Arabian Peninsula

Although until recent times the majority of the Saudi Arabia population was nomad, a moderate level of mitochondrial genetic structure has been found amongst its different regions. This heterogeneity grew considerably when all the Arabian Peninsula countries were included in the AMOVA analysis. It seems that the main cause of this diversity is the unequal influence that the different areas received from their geographically closest neighbors. This fact is graphically reflected in the MDS plot (Figure 6) where all the Arabian Peninsula samples are compared with samples from East Africa, the Near East and the Caucasus areas. The clustering of all the Saudi regions clearly shows that, in comparison to other geographically more distant populations, they form a rather homogenous entity, as was previously suggested from analysis based on classical markers [12]. The more distant positions of Yemen, grouped with African samples, and the UAE, and in a lesser degree the Qatar and Oman, proximity to Near East countries, reflect their different frequencies of African and Eurasian lineages in their respective mitochondrial pools. Roughly, the African contribution to whole Arabian Peninsula accounts for 20% of its lineages if, in addition to all the L haplogroups, the North African M1 and U6 clades are added. However, the western and southern areas have received significantly stronger influences than the rest. Particularly, Yemen has the largest contribution of L lineages [30]. So, most probably, this area was the entrance gate of a portion of these lineages in prehistoric times, which participated in the building of the primitive Arabian population. Later, received gene flows from North Africa and the Near East, and suffered expansions and retractions in humid or arid climatic periods. These fluctuations are also reflected in the frequent loss of diversity for several African clades as the L6 in Yemen [30] or the L5 in Saudi Arabia. However, the presence of western Africa L lineages and the different composition of L subclades in the African pool of different countries might reflect unequal participation of the primitive and the recent slave trade substrates in their respective African components.

An important group of the Arabian Peninsula lineages (18%), comprising representatives of the majority of the U clades, R2, and Central Asian, Indian, and Indonesian M lineages, seem to have their origins in the East, reaching the Arabian Peninsula through Iran where, in contrast to the Near East, the U clades (29%) have the highest frequency instead of the H (17%) group [49]. Congruently, this Eastern gene flow had a significantly stronger impact in the Eastern and Southern areas of the Arabian Peninsula. However, the bulk of the Arabian N and R lineages (62%) had a Northern source. Haplogroups (preHV)1 and J1b were the main contributors of this gene flow. Nevertheless, its present day geographic distributions in the Arabian Peninsula are different. Whereas (preHV)1 presents significant higher frequencies in the North and Central Saudi regions and in Oman, J1b shows its highest frequencies in the more peripheral West and Southeast Saudi regions. It seems that at least haplogroups H, N1c and subclade T3 could have followed the (preHV)1 internal way of dispersion, while the T1 and T5 branches of haplogroup T and other branches of haplogroup J followed the peripheral route of clade J1b. Attending to the radiation ages of (preHV)1 and J1b clades and their derivate branches, striking similarities but also differences can be observed. The first expansion of both clades in the Near East had similar Paleolithic ages around 20,000 years ago. However whereas the ancestral HVSI motif of the (preHV)1 expansion was barely present in Saudi Arabia [31], the ancestral HVSI motif of the J1b radiation had an important incidence in that area (Figure 5) suggesting an active role in Arabia of the first J1b spread but not for that of (preHV)1. The succeeding most important radiations of both clades, (preHV)1a1 and J1b1a1 had, again, similar ages around 10,000 years that place them in Neolithic times. Now, in both cases, there is a shortage or absence of the ancestral motif in Arabia discarding this area as a radiation center. However, it participated in the (preHV)1a1 spread [31] but not in the J1b1a1 one (Figure 5). Finally, the third more abundant subclades, (preHV)1b rooted by 16304 [31] and J1b rooted by 16136 (Figure 5) had the Arabian Peninsula as the most probable source of expansion. Nevertheless, whereas the J1b branch TMRCA (11,099 ± 8,381 years ago) was contemporary to that of the northern J1b1a1, the recalculated age of the (preHV)1b branch (by adding all the new HVSI sequences found in the present survey to the ones previously used [31]), was of only 4,036 ± 2,211 years ago which situates this expansion in the Bronze Age. These results could be satisfactorily explained if we admit an older Paleolithic implantation in Saudi Arabia of the J1b clade that, perhaps, with some other N and L clades would form the primitive population. Posterior (preHV)1 subclade radiations, accompanied by other clades, penetrated from the North using internal routes and even had secondary spreads in central Arabia diluting the J1b frequencies in these areas and causing its peripheral distribution.

### Genomic dissection of rare M lineages

By genomic sequencing of seven M lineages (Accession numbers: EU370391–97), it has been demonstrated that the majority of the rare M lineages detected in Saudi Arabia (Figure 3) have Indian roots. However, the link found between the M Saudi 201 sequence and an M14 Australian sequence is puzzling. Although at first sight it could be taken as a signal of the connection between the two utmost ends of the southern route, it seems not to be the case. First, both lineages share three basal positions and this hypothetical link would considerably delay the arrival age of M in comparison to that of East Asia. It would be improbable that similar Australian links with other M lineages mainly from India were not found. Third, if the Arab lineage had such an old implantation in the Arabian Peninsula some detectable autochthonous radiation should be expected. Most probably, the M42 sequence belongs to an Australian clade and its related lineage found in Saudi Arabia is also of Australian origin. Historical links as those invoked to explain the presence of Indian and Indonesian sequences in the Arabian Peninsula pool should also be valid for this case. In our opinion, the camel trade between Saudi Arabia and Australia [54] could be a probable historic cause of this link. Future detection in Aboriginal Australians of other M42 lineages will confirm the Australian origin of this clade and its radiation age in that Continent. However, the link between the East Asia M10 clade [40] and the Australian M42 clade, if not due to convergence, seems to be more interesting as it would confirm, once more, the rapid expansion of macrohaplogroup M all along the Asian coasts [6,13]. The lack of autochthonous M and N lineages in the present day Arabian Peninsula populations confirms that this area was not a place of demographic expansion in the dispersal out of Africa [55].

## Conclusion

Although there is evidence of Neolithic and more recent expansions in the Arabian Peninsula, mainly detected by (preHV)1 and J1b lineages, the lack of primitive autochthonous M and N sequences, suggests that this area has been more a receptor of human migrations, including historic ones, from Africa, India, Indonesia and even Australia, than a demographic expansion center along the proposed southern coastal route.

## Methods

### Study population

Buccal swabs or peripheral blood were obtained from 553 (120 of them previously published in Abu-Amero et al. [31]) maternally unrelated Saudi Arabs all whose known ancestors were of Saudi Arabian origin. The main Saudi Arabian geographic regions were sampled (Figure 1 and Additional file 1). Sequence analysis was performed of mtDNA regulatory region hypervariable segment I (HVSI) and hypervariable segment II (HVSII) and of haplogroup diagnostic mutations using RFLPs or partial sequencing [see Additional file 1]. In addition, genomic mtDNA sequencing was carried out in 7 individuals of uncertain or interesting haplogroup adscription. For population and phylogeographic comparison, we used 21,808 published or unpublished partial sequences from Europe (11,174), South Asia (2,746), Caucasus (1,638), North Africa (1,009), East Africa (888), Near East (2,001), Arabian Peninsula (1,129) and Jews (1,223), as detailed in Additional files 4 and 5. Informed consent was obtained from all individuals.

### MtDNA sequencing

Total DNA was isolated from buccal and blood samples using the PUREGENE DNA isolation kit from Gentra Systems (Minneapolis, USA). HVSI and HVSII segments were PCR amplified using primers pairs L15840/H16401 and L16340/H408, respectively, as previously described [6]. Genomic mtDNA sequences and segments including diagnostic positions were amplified using a set of 32 separate PCRs and cycling conditions as detailed elsewhere [6]. Successfully amplified products were sequenced for both complementary strands using the DYEnamic™ ET dye terminator kit (Amersham Biosciences), and samples were run on MegaBACE 1000 (Amersham Biosciences) according to the manufacturer protocol.

### Haplotype classification

Classification into sub-haplogroups was performed using nomenclature previously described for African [35,30,34] and for Eurasian [47,49,40,24,20,37,31,26] sequences. Published sequences employed for comparative genetic analysis were re-classified into sub-clades using the same criteria in order to permit comparisons.

### Genetic analysis

Haplotype diversity was calculated as h [56] and as K (haplotype number/sample size quotient). Only HVSI positions from 16069 to 16385 were used for genetic comparisons of partial sequences with other published data. Genetic variation was apportioned within and among geographic regions using AMOVA by means of ARLEQUIN2 [57]. Four regions (North, Central, West and South-East) were considered to assess the Saudi Arabia genetic structure (Figure 1 and Additional file 2). For more extended geographic comparisons the following areas were taken into account: Arabian Peninsula (including Saudi Arabia, Qatar, UAE, Oman, Yemen and Bedouin Arabs), North-East Africa (including samples from Egypt, Nubian, Sudan, Ethiopia, and Kenya), and Near East (containing samples from Druze, Iran, Iraq, Jordan, Kurds, Palestine, Syria and Turkey), as detailed in Additional file 3. Pairwise F_ST _distances between populations were calculated from haplogroup and haplotype frequencies, and their significance assessed by a nonparametric permutation test (ARLEQUIN2). Multidimensional scaling (MDS) plots were obtained with SPSS version 13.0 (SPSS Inc., Chicago, Illinois). Phylogenetic relationships among HVSI and genomic mtDNA sequences were established using the reduced median network algorithm [58]. In addition to our 7 genomic mtDNA sequences, 7, 12 and 23 published complete or nearly complete mtDNA sequences were used to establish the M1 (Figure 2), M (Figure 3) and J1b (Figure 4) phylogenies, respectively.

### Time estimates

Only substitutions in the coding region were taken into account for complete sequences, excluding insertions and deletions. The mean number of substitutions per site compared to the most common ancestor (ρ) of each clade and its standard error were calculated following Morral et al. [59] and Saillard et al. [60] respectively, and converted into time using previously published substitution rates [5,52]. For HVSI, the age of clusters or expansions was calculated as the mean divergence (ρ) from inferred ancestral sequence types [59,60] and converted into time by assuming that one transition within np 16090–16365 corresponds to 20,180 years [61].

## Accession numbers

The seven new complete mitochondrial DNA sequences are registered under GenBank accession numbers: EU370391–EU370397

## Authors' contributions

KKAA, JML, VMC and AMG conceived and designed the experiments and wrote the paper. KKAA and VMC performed the experiments. JML and AMG analyzed the data. All the authors read and approved the final manuscript.

## Supplementary Material

Additional File 1 HVS I and II sequences and RFLP results for the Saudi Arabs analyzed. The data provided shows the ID, geographic localization, HVS I and II sequence, and RFLPs for each individual analyzed. Click here for file

Additional File 2 Haplogroup frequencies (in percentage) and sample size for the different Saudi Arab regions. The data show the sample size and haplogroup frequencies (in percentage) for each of the different regions (central, north, southeast, west, and miscellaneous sample) analyzed in Saudi Arab. Click here for file

Additional File 3 Sample size and frequency (in percentage) of different groups in the populations studied, used to estimate the FST between them. The data show the sample size, and haplogroup frequencies (in percentage) for the following studied populations: Saudi Arab, Yemen, Oman, Qatar, United Arab Emirates, Bedouins, Iran, Iraq, Syria, Palestine, Jordan, Druze, Turkey, Kurd, Egypt, Kenya, Nubian, Sudan, and Ethiopia. Click here for file

Additional File 4 Populations searched for J1b haplotypes. The table lists the populations and geographic areas used to search for J1b haplotypes. Sample sizes and references for each population are also indicated. Click here for file

Additional File 5 References used in Additional file 4. References cited in Additional file 4 are detailed. Click here for file
