# Supplementary material for: Mitochondrial DNA structure in the Arabian Peninsula
Source: BMC Evol Biol. 2008 Feb 12;8:45. doi: 10.1186/1471-2148-8-45 (PMC2268671; doi:10.1186/1471-2148-8-45)
Supplement: Additional File 5 — References used in Additional file 4. References cited in Additional file 4 are detailed. [file 1471-2148-8-45-S5.doc]

**Additional file 5. References used in Additional file 4**

Abu-Amero KK, González AM, Larruga JM, Bosley TM, VMCabrera. 2007. Eurasian and African mitochondrial DNA influences in the Saudi Arabian population. BMC Evolutionary Biology 7:32 doi:10.1186/1471-2148-7-32

Al-Zahery N, Semino O, Benuzzi G, Magri C, Passarino G, Torroni A, Santachiara-Benerecetti AS. 2003. Y-chromosome and mtDNA polymorphisms in Iraq, a crossroad of the early human dispersal and of post-Neolithic migrations. Mol Phylogenet Evol 28(3):458-472

Babalini C, Martinez-Labarga C, Tolk HV, Kivisild T, Giampaolo R, Tarsi T, Contini I, Barac L, Janicijevic B, Martinovic Klaric I, Pericic M, Sujoldzic A, Villems R, Biondi G, Rudan P, Rickards O. 2005. The population history of the Croatian linguistic minority of Molise (southern Italy): a maternal view. Eur J Hum Genet 13(8):902-912

Behar DM, Hammer MF, Garrigan D, Villems R, Bonne-Tamir B, Richards M, Gurwitz D, Rosengarten D, Kaplan M, Pergola SD, Quintana-Murci L, Skorecki K. 2004. MtDNA evidence for a genetic bottleneck in the early history of the Ashkenazi Jewish population. Eur J Hum Genet 12(5):355-365

Belledi M, Poloni ES, Casalotti R, Conterio F, Mikerezi I, Tagliavini J, Excoffier L. 2000. Maternal and paternal lineages in Albania and the genetic structure of Indo-European populations Eur J Hum Genet 8(7):480-486

Bertranpetit J, Sala J, Calafell F, Underhill PA, Moral P, Comas D. 1995. Human mitochondrial DNA variation and the origin of the Basques. Ann Hum Genet 59:63-81

Bini C, Ceccardi S, Luiselli D, Ferri G, Pelotti S, Colalongo C, Falconi M, Pappalardo G. 2003. Different informativeness of the three hypervariable mitochondrial DNA regions in the population of Bologna (Italy). Forensic Sci Int 135(1):48-52

Bosch E, Calafell, González-Neira A, Flaiz C, Mateu E, Scheil HG, Huckenbeck W, Efremovska L, Mikerezi I, Xirotiris N, Grasa C, Schmidt H, Comas D. 2006. Paternal and maternal lineages in the Balkans show a homogeneous landscape over linguistic barriers, except for the isolated Aromuns. Ann Hum Genet 70:459-487

Brakez Z, Bosch E, Izaabel H, Akhayat O, Comas D, Bertranpetit J, Calafell F. 2001. Human mitochondrial DNA sequence variation in the Moroccan population of the Souss area. Ann Hum Biol 28(3):295-307

Brandstätter A, Peterson CT, Irwin JA, Mpoke S, Koech DK, Parson W, Parsons TJ. 2004. Mitochondrial DNA control region sequences from Nairobi (Kenya): inferring phylogenetic parameters fro the establishment of a forensic database. Int J legal Med 118:294-306

Calafell F, Underhill P, Tolun A, Angelicheva D, Kalaydjeva L. 1996. From Asia to Europe: mitochondrial DNA sequence variability in Bulgarians and Turks. Ann Hum Genet 60:35-49

Cali F, Le Roux MG, D'Anna R, Flugy A, De Leo G, Chiavetta V, Ayala GF, Romano V.J. 2001. MtDNA control region and RFLP data for Sicily and France. Int J Legal Med 114(4-5):229-231

Casas MJ, Hagelberg E, Fregel R, Larruga JM, González AM. 2006. Human mitochondrial DNA diversity in an archaeological site in *al-Andalus*. Genetic impact of migrations from North Africa in medieval Spain. Am J Phys Anthropol 131:539-551

Cherni L, Loueslati BY, Pereira L, Ennafaa H, Amorim A, El Gaaied AB. 2005. Female gene pools of Berber and Arab neighboring communities in central Tunisia: microstructure of mtDNA variation in North Africa. Hum Biol 77(1):61-70

Comas D, Calafell F, Mateu E, Perez-Lezaun A, Bertranpetit J. 1996.Geographic variation in human mitochondrial DNA control region sequence: the population history of Turkey and its relationship to the European populations. Molec Biol Evol 13:1067-1077

Comas D, Calafell F, Mateu E, Perez-Lezaun A, Bosch E, Martinez-Arias R, Clarimon J, Facchini F, Fiori G, Luiselli D, Pettener D, Bertranpetit J. 1998. Trading genes along the silk road: mtDNA sequences and the origin of central Asian populations. Am J Hum Genet 63(6):1824-1838

Comas D, Calafell F, Bendukidze N, Fananas L, Bertranpetit J. 2000.Georgian and kurd mtDNA sequence analysis shows a lack of correlation between languages and female genetic lineages. Am J Phys Anthropol 112(1):5-16

Comas D, Plaza S, Wells RS, Yuldaseva N, Lao O, Calafell F, Bertranpetit J. 2004. Admixture, migrations, and dispersals in Central Asia: evidence from maternal DNA lineages.Eur J Hum Genet. 12(6):495-504

Corte-Real HB, Macaulay VA, Richards MB, Hariti G, Issad MS, Cambon-Thomsen A, Papiha S, Bertranpetit J, Sykes BC. 1996. Genetic diversity in the Iberian Peninsula determined from mitochondrial sequence analysis. Ann Hum Genet 60:331-350

Crespillo M, Luque JA, Paredes M, Fernández R, Ramírez E, Valverde JL. 2000. Mitochondrial DNA sequences for 118 individuals from northeastern Spain. Int J Legal Med 114:130-132

Di Rienzo A, Wilson AC. 1991.Branching pattern in the evolutionary tree for human mitochondrial DNA. Proc Nat Acad Sci USA 88:1597-1601

Di Benedetto G, Erguven A, Stenico M, Castri L, Bertorelle G, Togan I, Barbujani G. 2001. DNA diversity and population admixture in Anatolia. Am J Phys Anthropol 115(2):144-156

Dubut V, Chollet L, Murail P, Cartault F, Beraud-Colomb E, Serre M, Mogentale-Profizi. 2004. DNA polymorphisms in five French groups: importance of regional sampling.Eur J Hum Genet 12(4):293-300

Fadhlaoui-Zid K, Plaza S, Calafell F, Ben Amor M, Comas D, Bennamar El gaaied A. 2004. Mitochondrial DNA heterogeneity in Tunisian Berbers. Ann Hum Genet 68 (3):222-233

Falchi A, Giovannoni L, Calo CM, Piras IS, Moral P, Paoli G, Vona G, Varesi L. 2006. Genetic history of some western Mediterranean human isolates through mtDNA HVR1 polymorphisms. J Hum Genet 51(1):9-14

Forster P, Cali F, Rohl A, Metspalu E, D'Anna R, Mirisola M, De Leo G, Flugy A, Salerno A, Ayala G, Kouvatsi A, Villems R, Romano V. 2002. Continental and subcontinental distributions of mtDNA control region types Int J Legal Med 116(2):99-108

González AM, Brehm A, Perez JA, Maca-Meyer N, Flores C, Cabrera VM. 2003. Mitochondrial DNA affinities at the Atlantic fringe of Europe. Am J Phys Anthropol 120(4):391-404

González AM, Cabrera, VM, Larruga JM, Tounkara A, Noumsi G, Thomas B, Moulds JM. 2006. Mitochondrial DNA variation in Mauritania and Mali and their genetic relationship to other western Africa populations. Ann Hum Genet 70: 631-657

González et al., unpublished

Helgason A, Hickey E, Goodacre S, Bosnes V, Stefansson K, Ward R, Sykes B. 2001. mtDna and the islands of the North Atlantic: estimating the proportions of Norse and Gaelic ancestry. Am J Hum Genet 68(3):723-737

Hofmann S, Jaksch M, Bezold R, Mertens S, Aholt S, Paprotta A, Gerbitz KD. Population genetics and disease susceptibility: characterization of central European haplogroups by mtDNA gene mutations, correlation with D loop variants and association with disease. Hum Mol Genet. 1997. 6(11):1835-46.

Kivisild T, Reidla M, Metspalu E, Rosa A, Brehm A, Pennarun E, Parik J, Geberhiwot T, Usanga E, Villems R. 2004. Ethiopian Mitochondrial DNA Heritage: Tracking Gene Flow Across and Around the Gate of Tears. Am J Hum Genet 75:752-770

Kouvatsi A, Karaiskou N, Apostolidis A, Kirmizidis G. 2001. Mitochondrial DNA sequence variation in Greeks. Hum Biol 73(6):855-869

Krings M, Halim Salem A, Bauer K, Geisert H, Malek AK, Chaix L, Simon C, Welsby D, Di Rienzo A, Utermann G, Sajantila A, Pääbo S, Stoneking M. 1999. mtDNA analysis of Nile valley populations: A genetic corridor or a barrier to migration? Am J Hum Genet 64(4):1166-1176

Larruga JM, Diez F, Pinto FM, Flores C, González AM. 2001. Mitochondrial DNA characterisation of European isolates: the Maragatos from Spain. Eur J Hum Genet 9(9):708-716

Lutz S, Weisser H-J, Heizmann J, Pollak S. Location and frequency of polymorphic positions in the mtDNA control region of individuals from Germany. 1998. Int J Legal Med 111:67-77

Maca-Meyer N, Sánchez-Velasco P, Flores C, Larruga JM, González AM, Oterino A, Leyva-Cobian F. 2003. Y chromosome and mitochondrial DNA characterization of Pasiegos, a human isolate from Cantabria (Spain). Ann Hum Genet 67:329-339

Macaulay V, Richards M, Hickey E, Vega E, Cruciani F, Guida V, Scozzari R, Bonné-Tamir B, Sykes B, Torroni A. 1999. The emerging tree of West Eurasian mtDNAs: a synthesis of control-region sequences and RFLPs. Am J Hum Genet 64: 232-249

Malyarchuk BA, Grzybowski T, Derenko MV, Czarny J, Wozniak M, Miscicka-Sliwka D. 2002. Mitochondrial DNA variability in Poles and Russians. Ann Hum Genet 66:261-283

Malyarchuk BA, Grzybowski T, Derenko MV, Czarny J, Drobnic K, Miscicka-Sliwka D. 2003. Mitochondrial DNA variability in Bosnians and Slovenians.Ann Hum Genet 67:412-425

Martinez-Jarreta B, Prades A, Calafell F, Budowle B. Mitochondrial DNA HVI and HVII variation in a north-east Spanish population. Forensic Sci 2000. 45(5):1162-3

McEvoy B, Richards M, Forster P, Bradley DG. 2004. The Longue Duree of genetic ancestry: multiple genetic marker systems and Celtic origins on the Atlantic facade of Europe. Am J Hum Genet 75(4):693-702

Mergen H, Öner R, Öner C. 2004. Mitochondrial DNA sequence variation in the Anatolian Peninsula (Turkey). J Genet 83(1):101-109

Metspalu M, Kivisild T, Metspalu E, Parik J, Hudjashov G, Kaldma K, Serk P, Carmín M, Behar DM, Gilbert MTP, Endicott P, Mastana S, Papiha SS, Skorecki K, Torroni A,Villems R. 2004. Most of the extant mtDNA boundaries in South and Southwest Asia were likely shaped during the initial settlement of Eurasia by anatomically modern humans BMC Genet 2004; 5: 26

Nasidze I, Stoneking M. 2001. Mitochondrial DNA variation and language replacements in the Caucasus. Proc R Soc Lond 268:1197-1206

Nasidze I, Ling ES, Quinque D, Dupanloup I, Cordaux R, Rychkov S, Naumova O, Zhukova O, Sarraf-Zadegan N, Naderi GA, Asgary S, Sardas S, Farhud DD, Sarkisian T, Asadov C, Kerimov A, M. Stoneking M. 2004. Mitochondrial DNA and Y-chromosome variation in the Caucasus. Ann Hum Genet 68: 205-221

Nasidze I, Quinque D, Dupanloup I, Rychkov S, Naumova O, Zhukova O, Stoneking M. 2004. Genetic evidence concerning the origins of South and North Ossetians. Ann Hum Genet 68: 588-599

Nasidze I, Quinque D, Ozturk M, Benndukidze N, Stoneking M. 2005. MtDNA and Y-chromosome variation in the Kurdish groups. Ann Hum Genet 69:401-412

Opdal SH, Rognum TO, Vege A, Stave AK, Dupuy BM, Egeland T. 1998. Increased number of substitutions in the D-loop of mitochondrial DNA in the sudden infant death syndrome. Acta Paediatr 87(10):1039-1044

Parson W, Parsons TJ, Scheithauer R, Holland MM. 1998. Population data for 101 Austrian Caucasian mitochondrial DNA d-loop sequences: application of mtDNA sequence analysis to a forensic case. Int J Legal Med 111:124-132

Pereira L, Cunha C, Amorim A. 2004. Predicting sampling saturation of mtDNA haplotypes: an application to an enlarged Portuguese database. Int J Legal Med 118(3):132-136

Picornell A, Gomez-Barbeito L, Tomas C, Castro JA, Ramon MM. 2005. Mitochondrial DNA HVRI variation in Balearic populations. Am J Phys Anthropol 128(1):119-130

Piercy R, Sullivan K, Benson N, Gill P. 1993. The application of mitochondrial DNA typing to the study of white Caucasian genetic identification. Int J Leg Med 106:85-90

Plaza S, Calafell F, Helal A, Bouzerna N, Lefranc G, Bertranpetit J, Comas D. 2003. Joining the pillars of Hercules: mtDNA sequences show multidirectional gene flow in the western Mediterranean. Ann Hum Genet 67:312-328

Poetsch M, Wittig H, Krause D, Lignitz E. 2003.Mitochondrial diversity of a northeast German population sample. Forensic Sci Internat 137(2-3):125-132

Pult I, Sajantila A, Simanainem J, Georgiev O, Schaffner W, Paabo S. 1994. Mitochondrial DNA sequences from Switzerland Reveal striking Homogeneity of European Populations. Biol Chen 375:837-840

Quintana-Murci L, Chaix R, Wells RS, Behar DM, Sayar H, Scozzari R, Rengo C, Al-Zahery N, Semino O, Santachiara-Benerecetti AS, Coppa A, Ayub Q, Mohyuddin A, Tyler-Smith C, Qasim Mehdi S, Torroni A, McElreavey K. 2004. Where west meets east: the complex mtDNA landscape of the southwest and Central Asian corridor. Am J Hum Genet 74(5):827-845

Rando JC, Pinto F, González AM, Hernández M, Larruga JM, Cabrera VM, Bandelt HJ. 1998. Mitochondrial DNA analysis of northwest African populations reveals genetic exchanges with European, near-eastern, and sub-Saharan populations. Ann Hum Genet 62:531-550

Rando JC, Cabrera VM, Larruga JM, Hernández M, González AM, Pinto F, Bandelt HJ. 1999. Phylogeographic patterns of mtDNA reflecting the colonization of the Canary Islands.Ann Hum Genet 63:413-428

Richards M, Corte-Real H, Forster P, Macaulay V, Wilkinson-Herbots H, Demaine A, Papiha S, Hedges R, Bandelt HJ, Sykes B. 1996. Paleolithic and Neolithic lineages in the European mitochondrial gene pool. Am J Hum Genet 59:185-203

Richards M, Macaulay V, Hickey E, Vega E, Sykes B, Guida V, Rengo C, Sellitto D, Cruciani F, Kivisild T, Villems R, Thomas M, Rychkov S, Rychkov O, Rychkov Y, Gölge M, Dimitrov D, Hill E, Bradley D, Romano V, Cali F, Vona G, Demaine A, Papiha S, Triantaphyllidis C, Stefanescu G, Hatina J, Belledi M, Di Rienzo A, Novelletto A, Oppenheim A, Norby S, Al-Zaheri N, Santachiara-Benerecetti S, Scozari R, Torroni A, Bandelt HJ. 2000. Tracing European founder lineages in the Near Eastern mtDNA pool.Am J Hum Genet 67(5):1251-1276

Rousselet F, Mangin P. 1998. Mitochondrial DNA polymorphisms: a study of 50 French Caucasian individuals and application to forensic casework. Int J Legal Med 111(6):292-298

Rowold DJ, Luis JR, Terreros MC, Herrera RJ. 2007.Mitochondrial DNA geneflow indicates preferred usage of the Levant Corridor over the Horn of Africa passageway. J Hum Genet 52:436–447. DOI 10.1007/s10038-007-0132-7

Sajantila A, Lahermo P, Anttinen T, Lukka M, Cistonen P, Savontaus M-L, Aula P Beckman L, Tranebjaerg L, Gedde-Dahl T, Issel-Tarver L, DiRienzo A, Paabo S.. Genes and languages in Europe: an analysis of mitochondrial lineages.1995. Genome Res 5:42-52

Sajantila A, Salem AH, Savolainen P, Bauer K, Gierig C, Paabo S. 1996. Paternal and maternal DNA lineages reveal a bottleneck in the founding of the Finnish population. Proc Natl Acad Sci U S A 93(21):12035-12039

Santos C, Lima M, Montiel R, Angles N, Pires L, Abade A, Aluja MP. 2003. Genetic Structure and Origin of Peopling in the Azores Islands (Portugal): The view from mtDNA. Ann Hum Genet 67:433-456

Stevanovitch A, Gilles A, Bouzaid E, Kefi R, Paris F, Gayraud RP, Spadoni JL, El-Chenawi. 2003. Mitochondrial DNA Sequence diversity in a sedentary population from Egypt. Ann Hum Genet 68:23-39

Tagliabracci A, Turchi C, Buscemi L, Sassaroli C. 2001. Polymorphism of the mitochondrial DNA control region in Italians Int J Legal Med114(4-5):224-228

Thomas MG, Weale ME, Jones AL, Richards M, Smith A, Redhead N, Torroni A, Scozzari R, Gratrix F, Tarekegn A, Wilson JF, Capelli C, Bradman N, Goldstein DB. 2002. Founding mothers of Jewish communities: geographically separated Jewish groups were independently founded by very few female ancestors. Am J Hum Genet 70:1411-1420

Tolk HV, Barac L, Pericic M, Klaric IM, Janicijevic B, Campbell H, Rudan I, Kivisild T, Villems R, Rudan P. 2001. The evidence of mtDNA haplogroup F in a European population and its ethnohistoric implications. Eur J Hum Genet 9:717-723

Varesi L, Memmí M, Cristofari M-C, Mameli GE, Caló CM, Vona G. 2000. Mitochondrial control-region sequence variation in the Corsican population, France. Am J Hum Biol12:339-351

Verginelli F, Donati F, Coia V, Boschi I, Palmirotta R, Battista P, Costantini RM, Destro-Bisol G. 2003. Variation of the hypervariable region-1 of mitochondrial DNA in central-eastern Italy. J Forensic Sci 48(2):443-444

Vernesi C, Di Benedetto G, Caramelli D, Secchieri E, Simoni L, Katti E, Malaspina P, Novelletto A, Marin VTM, Barbujani G. 2001. Genetic characterization of the body attributed to the evangelist Luke. PNAS 98(23):13460-13463

Vona G, Ghiani ME, Caló CM, Vacca L, Memmí M, Varesi L. 2001. Mitochondrial DNA variation sequence análisis in Sicily. Am J Hum Biol 13:576-589

Zupanic Pajnic I, Balazic J, Komel R. 2004. Sequence polymorphism of the mtDNA control region in the Slovenian population. Int J Legal Med 118:1-4
